# Extracellular matrix expansion by cardiac magnetic resonance T1 mapping- validation with myocardial biopsy

**DOI:** 10.1186/1532-429X-17-S1-P308

**Published:** 2015-02-03

**Authors:** Andreas Kammerlander, Caroline Tufaro, Alina F Bachmann, Beatrice A Marzluf, Stefan Aschauer, Andreas Greiser, Diana Bonderman, Julia Mascherbauer

**Affiliations:** 1 Medical University of Vienna, Vienna, Austria; 2 Siemens AG Healthcare Sector, Erlangen, Germany

## Background

Accumulation of diffuse myocardial fibrosis / extracellular volume (ECV) in the myocardium is common to various cardiac diseases and is associated with an unfavorable prognosis. Various cardiac magnetic resonance (CMR) T1 mapping techniques have recently been proposed for the quantification of ECV, including:

1. Modified Look-Locker Inversion recovery (MOLLI) T1 mapping including calculation of ECV, 2. Post-contrast multiple-breath-hold T1 mapping. 3. Native (pre-contrast) T1 mapping.

T1 mapping is a promising technique, however, validation data based on myocardial biopsy are sparse.

## Methods

36 heart failure patients underwent CMR T1 mapping and left-ventricular biopsy within 4 weeks. CMR protocols (1.5-T scanner, (Magnetom Avanto, Siemens Healthcare, Erlangen, Germany) included non-product MOLLI and multiple-breath-hold T1 mapping.

The population consisted of 22 patients with HFpEF (heart failure with preserved ejection fraction), 7 with cardiac amyloidosis, 3 with HFrEF (heart failure with reduced ejection fraction) and 4 with organic mitral regurgitation (MR).

Specimens were stained using modified Trichrome. TissueFAXS and dedicated software were used to quantify ECV.

## Results

ECV by TissueFAXS was 32±17% and 35±13% by CMR MOLLI. Post-contrast T1 times by the multiple-breath-hold sequence were 411±79ms, native T1 times were 1009±73ms. Patients with amyloidosis had significantly more ECV by TissueFAXS (59±13%, p<0.001) and with CMR MOLLI (56±12%, p<0.001) compared with the other pathologies.

The amount of TissueFAXS ECV correlated significantly with native T1 times (r=0.657, p<0.001), MOLLI ECV (r=0.907, p<0.001) and with multiple-breath-hold post-contrast T1 times (r=-0.683, p<0.001). When excluding patients with amyloidosis, only MOLLI ECV significantly correlated with histology (r=0.491, p=0.015), whereas multiple-breath-hold post-contrast T1 times just missed the level of significance (r=-0.413, p=0.056).

## Conclusions

In our series, MOLLI ECV appears to be the most promising method for the quantification of extracellular matrix when validated against histology. Future studies need to define the prognostic value of T1 mapping in a large prospective cohort.

## Funding

The study received support from the Österreichischer Herzfonds and the Austrian fellowship grants KLI 245.

